# Executive functioning and neurodevelopmental disorders in early childhood: a prospective population-based study

**DOI:** 10.1186/s13034-019-0299-7

**Published:** 2019-10-22

**Authors:** D. Louise Otterman, M. Elisabeth Koopman-Verhoeff, Tonya J. White, Henning Tiemeier, Koen Bolhuis, Pauline W. Jansen

**Affiliations:** 1 Department of Child and Adolescent Psychiatry/Psychology, Erasmus MC-University Medical Center-Sophia Children’s Hospital, Wytemaweg 80, 3000 CA Rotterdam, The Netherlands; 2 The Generation R Study Group, Erasmus Medical Center, Rotterdam, The Netherlands; 3 Department of Psychiatry and Human Behavior, Alpert Medical School of Brown University, Providence, RI USA; 4 Department of Radiology, Erasmus University Medical Center, Rotterdam, The Netherlands; 5 Department of Social and Behavioral Science, Harvard TH Chan School of Public Health, Boston, MA USA; 6 Department of Psychiatry, Amsterdam UMC, University of Amsterdam, Amsterdam, The Netherlands; 7 Department of Psychology, Education and Child Studies, Erasmus University Rotterdam, Rotterdam, The Netherlands

**Keywords:** Executive functioning, Autism, ADHD, Population-based, Longitudinal

## Abstract

**Background:**

Executive functioning deficits are common in children with neurodevelopmental disorders. However, prior research mainly focused on clinical populations employing cross-sectional designs, impeding conclusions on temporal neurodevelopmental pathways. Here, we examined the prospective association of executive functioning with subsequent autism spectrum disorder (ASD) traits and attention-deficit/hyperactivity disorder (ADHD) traits.

**Methods:**

This study included young children from the Generation R Study, a general population birth cohort. The Brief Rating Inventory of Executive Function-Preschool Version was used to assess parent-reported behavioral executive functioning when the children were 4 years old. ASD traits were assessed at age 6 (n = 3938) using the parent-reported Social Responsiveness Scale. The Teacher Report Form was used to assess ADHD traits at age 7 (n = 2749). Children with high scores were screened to determine possible clinical ASD or ADHD diagnoses. We were able to confirm an ASD diagnosis for n = 56 children by retrieving their medical records and established an ADHD diagnosis for n = 194 children using the Diagnostic Interview Schedule for Children-Young Child version (DISC-YC). Data were analyzed using hierarchical linear and logistic regressions.

**Results:**

Impaired executive functioning was associated with more ASD and ADHD traits across informants (for ASD traits and diagnoses: β = 0.33, 95% CI [0.30–0.37]; OR = 2.69, 95% CI [1.92–3.77], respectively; for ADHD traits and diagnoses: β = 0.12, 95% CI [0.07–0.16]; OR = 2.32, 95% CI [1.89–2.85], respectively). Deficits in all subdomains were associated with higher levels of ASD traits, whereas only impaired inhibition, working memory, and planning/organization were associated with more ADHD traits.

**Conclusions:**

The findings of the current study suggest a graded association of executive functioning difficulties along the continuum of ASD and ADHD and that problems in executive functioning may be a precursor of ASD and ADHD traits from an early age onwards.

## Background

Executive functions are a set of cognitive abilities that are needed for regulating behavior, including inhibition, working memory, and planning. The ability to regulate behavior is important, as executive functioning has a substantial impact on short-term and long-term life outcomes such as physical and mental health, performance in school, and socioeconomic status [1, 2]. Executive functioning is often impaired in psychiatric disorders [3, 4], including neurodevelopmental disorders, such as autism spectrum disorder (ASD) and attention-deficit/hyperactivity disorder (ADHD) [5, 6]. So far, little is known about early executive functioning problems in young children with subclinical traits of ASD and ADHD.

Autism spectrum disorder is characterized by deficits in social interaction and communication, and restricted behavior and interests, whereas the main symptoms in ADHD are inattention and hyperactivity/impulsivity [7]. The prevalence of these disorders among children under 18 years are approximately 1% [8, 9] and 3–5% [10, 11], respectively. Children with ASD and ADHD can have lower educational achievements and poorer social outcomes, with problems often extending into adulthood [12, 13]. Importantly, traits of ASD and ADHD occur along a continuum of severity [14, 15], ranging from sub-clinical to severely impaired. However, children with lower levels of ASD and ADHD traits, not sufficient for a diagnosis, are also suffering from daily impairments.

Executive functioning deficits associated with both ASD and ADHD are found consistently throughout the literature [5, 6, 16, 17]. The main domains in children with ASD comprise shifting, planning, and working memory [5, 6, 16], although broader executive functioning deficits across all domains have been observed as well [5, 18–20]. Conversely, children with ADHD have more pronounced difficulties in executive functioning, in the domains of inhibition, working memory, vigilance, and planning [5, 17, 18]. These difficulties are not only seen among those with a clinical diagnosis, as few population-based studies suggest that (young) children and adults with subclinical traits of ASD or ADHD also experience problems in executive functioning [21–26]. These findings are important, as children with subclinical traits of disorders often remain undetected by mental health services for various reasons [27–29], including symptoms not being severe enough to warrant help seeking, stigmatization of seeking help for mental problems, and inability to pay. However, sub-clinical symptoms may be associated with other sub-clinical characteristics, such as cognition function, which may result in some impairment [27, 30, 31]. Indeed, executive functioning has a substantial impact on short-term and long-term life outcomes [1, 2, 32].

Only a minority of studies in this field has focused on young children with neurodevelopmental traits. Young children with ADHD or at high risk for ADHD appear to be impaired in executive functioning [33–35], while research on young children with ASD is more inconclusive [36–39]. Some studies find no differences in executive functioning between children with and without ASD [38, 39], whereas others do, but depending on the different age or means of measuring executive functioning [20, 36, 37]. It has been argued that performance tasks and behavioral ratings should be distinguished from each other, as they may measure different aspects of executive functioning [40, 41]. Performance tasks are more situational and measure abilities in a specific (test-) environment, whereas behavioral ratings focus on the ability to apply these skills in daily life, perhaps making the latter more generalizable and therefore clinically more relevant.

Furthermore, most of the previous studies employed cross-sectional designs, impeding any conclusions on timing and temporality of associations. In addition, clinical studies often only include children in the clinical range, disregarding the other end of the spectrum. However, population studies include children from the general population, representing the full continuum and allowing for analysis along the entire dimension of executive functioning, ASD and ADHD. Potentially, deficits in executive functioning may be an expression of the latent vulnerability to ASD and ADHD [42]. A better understanding of neurodevelopmental pathways across early childhood may allow early identification and early intervention for children with traits of these disorders.

The aim of the current study was to investigate the association of executive functioning at age 4 years with ASD and ADHD traits at age 6/7 years. Specifically, we wanted to determine whether executive functioning could be an early indicator of later neurodevelopmental traits, independent of pre-existing traits. For this, we used a behavioral measure of executive functioning assessed in a general population cohort to explore impairment across the continuum of ASD and ADHD. Based on existing research, we expected impaired overall executive functioning to be prospectively associated with greater levels of ASD and ADHD traits. First, we expected that all executive functioning subdomains are associated with ASD traits. Second, we expect that specific executive function subdomains, including difficulties with inhibition, working memory, and planning, are associated with ADHD traits.

## Method

### Participants

This study was embedded in the Generation R Study [43], a large population-based prospective birth cohort in Rotterdam, the Netherlands. Pregnant women living in the study area with an expected delivery date between April 1, 2002 and January 31, 2006 were invited to participate. The overall response rate was 61%. The goal of the Generation R Study is to identify biological and environmental factors that influence growth, development, and health of children and their parents. A more detailed description of the cohort has been provided elsewhere [43]. The Medical Ethical Committee of the Erasmus Medical Center Rotterdam has approved the study. Written informed consent was obtained from all parents.

**Fig. 1:**
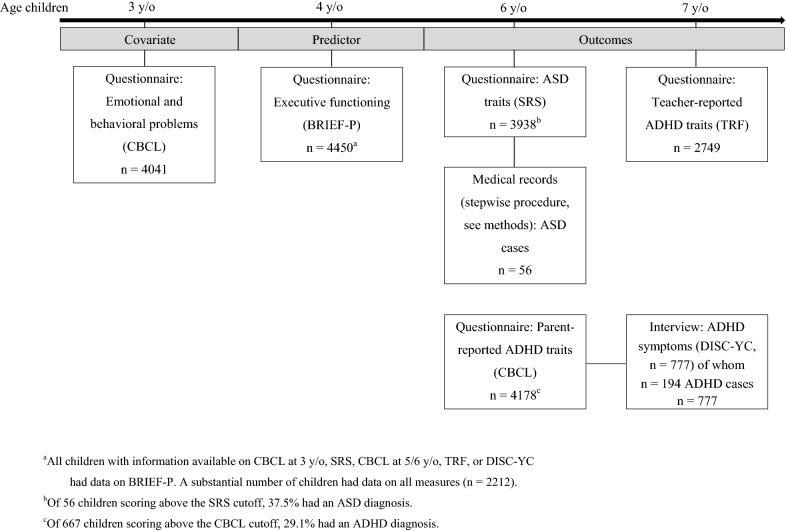
Population and measurements overview. *ADHD* attention-deficit/hyperactivity disorder, *ASD* autism spectrum disorder, *BRIEF-P* Brief Rating Inventory of Executive Functioning-Preschool version, *CBCL* Child Behavior Checklist, *SRS* Social Responsiveness Scale, *TRF* Teacher Report Form

In total, we had 4450 children in our sample whose parents all completed the executive functioning questionnaire and who had information available on at least one of the following three assessments: ASD traits as reported by parents (n = 3938), ADHD traits rated by the teacher (n = 2749), or ADHD symptoms acquired in a clinical interview conducted with parents (n = 777). Among these 4450 children were 56 with a clinician confirmed ASD diagnosis and 194 with an ADHD diagnosis established based on a clinical interview (see Fig. 1 for an overview of the study population and measures).

### Material

#### Executive functioning

At age 4 years (SD = 1 month), executive functioning was assessed with the validated Brief Rating Inventory of Executive Function-Preschool Version (BRIEF-P) [44–46]. The BRIEF-P was designed to measure executive functions in children aged 2 to 5 in everyday life. Parents (89% mothers) were asked to rate everyday executive functioning behavior of their children on a 3-point scale ranging from 1 (*never*) through 2 (*sometimes*) to 3 (*often*). Higher scores indicate more difficulties in executive functions. The BRIEF-P consists of 63 items covering five subscales: inhibition (16 items), shifting (10 items), emotional control (10 items), working memory (17 items), and planning/organization (10 items). All subscales and the total score were used in the analyses. Internal consistency of the overall score and the five dimensions was high: total score α = .95, inhibition α = .88, shifting α = .81, emotional control α = .83, working memory α = .89, planning/organization α = .78.

#### Child Behavior Checklist (CBCL)

The CBCL 1.5–5 is a screening measure for problems in young children, covering a wide range of emotional and behavioral problems, including pervasive developmental (i.e. ASD) and ADHD symptoms [47]. When the children were 3 (SD = 1.3 months) and 5/6 (SD = 3.8 months) years old, parents (100% and 91.9% mothers, respectively) completed the questionnaire. The CBCL 1.5–5 assessed at 3 years was used as a covariate in the analyses to adjust for baseline emotional and behavioral problems. The CBCL 1.5–5 at 5/6 years was part of the stepwise approaches to determine ASD and ADHD diagnoses. The questionnaire contains 99 items that are rated on a 3-point Likert scale, ranging from 0 (*not true*) to 2 (*very true* or *often true*), where higher scores indicate more problems. Here, we used the total problem score and the DSM-oriented ADHD subscale. The CBCL 1.5–5 has shown to be a reliable and valid measure for child emotional and behavioral problems [47] and is validated for use across 23 countries, including the Netherlands [48].

#### ASD traits

ASD traits were assessed when the children were 6 years of age (SD = 4.5 months) using the Social Responsiveness Scale (SRS) [49], which was completed by parents (92% mothers). The SRS is developed to measure clinical and subclinical ASD-like traits in children aged 4 to 18 years [49, 50]. In this study, an 18-item short form of the SRS was used to minimize the subject burden [51]. The short form covers the main criteria for an ASD diagnosis according to the *Diagnostic and Statistical Manual of Mental Disorders* (5th ed.; *DSM*-*V*) [7]. The items are rated on a 4-point Likert scale ranging from 0 (*never true*) to 3 (*almost always true*), with higher scores indicating more problems. Mean item scores were calculated by summing the items and dividing them by the number of endorsed items (25% missing values were allowed). The total score of the short form shows correlations of .93–.99 with the full scale in three different large studies [52] and showed good internal consistency in our sample (α = .78).

In addition to ASD traits measured with the SRS, cases with clinical ASD were identified [53]. Children with scores in the top 15th percentile of the total score or in the top 2nd percentile on the pervasive developmental disorder subscale of the CBCL 1.5–5 (assessed at age 5/6) were further screened with the Social Communication Questionnaire (SCQ), a 40-item measure for ASD that parents completed [54]. Screening of medical records for an ASD diagnosis was done for (1) children with scores of 15 or higher on the SCQ; (2) children who scored above the cutoff on the SRS (1.078 for boys and 1.000 for girls); and (3) children whose mothers reported at any moment before the age of 8 years that the child had undergone a diagnostic assessment for ASD. In the Netherlands, only licensed psychiatrists and psychologists are allowed to make clinical diagnoses. General practitioners hold an overview of all medical information about an individual, including mental health assessments. The general practitioners of children who met one or more of the three conditions were consulted to retrieve the medical records and check if a diagnosis had been made. Of 56 children scoring above the SRS cutoff, 37.5% had an ASD diagnosis, as confirmed by medical records.

#### ADHD traits

The Dutch version of the Teacher Report Form (TRF) 6–18 [55] was used to assess ADHD traits. The TRF 6–18 is the teacher version of the CBCL 6–18 and measures emotional and behavioral problems of children [56]. The TRF was administered to teachers when the children were 7 years old (SD = 1.2 years). The questionnaire contains 120 items that are rated on a scale from 0 (*not true*) through 1 (*sometimes true*) to 2 (*often true*), where higher scores indicate more problematic behavior. Only the DSM-oriented attention deficit hyperactivity problems subscale was used in this study. The scale comprises 13 items and had high internal reliability with a Cronbach’s alpha of .92.

Additionally, ADHD cases were identified using the Diagnostic Interview Schedule for Children-Young Child version (DISC-YC) [57, 58], which is the developmentally appropriate version of the DISC-parent version. It is a structured, clinical interview that assesses symptoms and impairment of disorders based on the DSM-IV in children 3–8 years of age. Trained interviewers administered the DISC-YC to parents during a home visit in a selection of our cohort when the children were on average 7 years old (SD = 0.7 years). Only children who had elevated scores on the CBCL 1.5–5 conducted at age 5/6 (top 15th percentile for total score or top 2nd percentile for any of the syndrome scales) were selected for an interview with the DISC-YC, as well as a random sample of children who scored under these cut-offs. The DISC-YC allows for identification of children who display all symptoms necessary for a clinical diagnosis based on the DSM-IV. Of 667 children scoring above the CBCL cutoff, 29.1% had an ADHD diagnosis, as established using the DISC-YC. In this study, we only used the diagnostic scale for ADHD, which has been shown to have good test–retest reliability [59].

#### Covariates

Multiple covariates were included in the analysis if they were likely to confound the relationship between executive functioning and ASD or ADHD traits. They were carefully selected based on prior research [60–62]. Gender and gestational age of the child were obtained from medical records, maintained by community midwives and hospitals. The country of birth of the parents defined child ethnic background. This was obtained through a questionnaire and divided into Dutch, other Western, and non-Western. Education of the mother was used as a measure of socio-economic status (SES). It was determined based on the highest completed education at the time the child was 5–6 years old and divided into three groups: low, middle, and high. Maternal psychopathology was assessed with the Dutch version of the Brief Symptom Inventory (BSI) [63] when the child was 3 years old. The four scales in this questionnaire were aggregated into a total psychopathology score, which was used in the analyses. Lastly, child emotional and behavioral problems at age 3 were measured with the CBCL 1.5–5 [47]. The total score was used in the analyses to account for any pre-existing psychopathology.

### Statistical analyses

Our aim was to examine the association of overall and subdomains of executive functioning with traits of ASD and ADHD. For each executive functioning subscale, we performed linear regression analyses. Logistic regression analyses were used to address the relationship of executive functioning with ASD and ADHD diagnoses. The regressions were performed in a hierarchical manner: the first model included the predictor only, covariates were added in the second model, and finally, in model 3, we additionally controlled for emotional and behavioral problems at age 3 years. This last step was included to be able to examine whether executive functioning deficits precede ASD and ADHD traits and to ensure that ADHD traits present at baseline could not explain the prospective association between executive functioning and ASD traits, and vice versa [64]. Lastly, to disentangle any potential differences between clinical and subclinical symptoms, sensitivity analyses were carried out, excluding children with an ASD or ADHD diagnosis from the analyses and rerunning the linear regression analyses [52].

We transformed non-normal variables prior to running the regression analyses with a square root transformation, including maternal psychopathology, baseline emotional and behavioral problems, all executive functioning variables, ASD traits, and ADHD traits. Missing values in the covariates were multiple imputed resulting in 10 imputed datasets.

## Results

Characteristics of the sample can be found in Table 1. The subsample with data available on ADHD traits (data not shown) had similar prevalence and mean levels of covariates as the sample with information on ASD traits. Children diagnosed with ASD (n = 56) or ADHD (n = 194) had higher levels of emotional and behavioral problems at age 3 years, executive functioning difficulties, ASD traits, and ADHD traits. Correlations between predictor and outcome variables can be found in Additional file 1: Table S1. Non-response analysis showed that children of non-Western ethnicity, children of mothers with lower education, and children with younger mothers were lost to follow up more often.

**Table 1:** Sample characteristics

	*n*	Sample with data on ASD traits *n* = 3938	*n*	ASD diagnoses sample *n* = 56	*n*	ADHD diagnoses sample *n* = 194
Child characteristics						
Gender (% boys)	3938	50.0	56	85.7	194	63.9
Gestational age at birth (weeks)	3926	39.85 (1.81)	56	39.17 (2.57)	194	39.74 (2.17)
Ethnicity	3933		56		194	
Dutch %	2771	70.5	43	76.8	121	62.4
Other Western %	358	9.1	3	5.4	20	10.3
Non-Western %	804	20.4	10	17.9	53	27.3
CBCL 1.5–5 total score	3665	18.11 (13.28)	51	29.91 (20.80)	176	32.10 (18.41)
BRIEF-P (executive functioning) total score	3901	85.28 (15.65)	56	108.07 (26.72)	189	104.35 (19.85)
Inhibition	3886	22.22 (5.09)	56	28.69 (7.55)	187	28.82 (6.39)
Shifting	3930	13.67 (3.34)	56	18.36 (5.67)	193	15.47 (4.34)
Emotional control	3932	14.24 (3.48)	56	18.20 (5.18)	193	17.27 (4.46)
Working memory	3892	21.55 (4.79)	56	27.01 (8.96)	191	26.38 (6.46)
Planning/organization	3927	13.61 (2.96)	56	15.80 (4.15)	192	16.42 (3.59)
SRS (ASD traits) score^a^	3938	0.21 (0.23)	54	0.94 (0.64)	169	0.50 (0.43)
TRF (ADHD traits) score	2272	3.00 (4.73)	34	7.50 (7.56)	116	6.97 (6.66)
Maternal characteristics						
Education level	3830		54		192	
Low %	76	2.0	1	1.9	10	5.2
Medium %	1153	30.1	22	40.7	73	38.0
High %	2601	67.9	31	57.4	109	56.8
BSI (psychopathology) score	3612	0.62 (1.01)	50	0.95 (1.29)	174	1.24 (1.60)

Values are mean total scores (standard deviation) unless stated otherwise

*ADHD* attention-deficit/hyperactivity disorder, *ASD* autism spectrum disorder, *BRIEF-P* Brief Rating Inventory of Executive Functioning-Preschool version, *BSI* Brief Symptom Inventory, *CBCL* Child Behavior Checklist, *SRS* Social Responsiveness Scale, *TRF* Teacher Report Form

^a^Mean item score. Sample with data on ADHD traits: n = 2749; overlap between sample with data on ASD traits and sample with data on ADHD traits: n = 2272

### Executive functioning and ASD traits

More executive functioning difficulties at age 4 were associated with higher levels of ASD traits at age 6 (β_adjusted_ = 0.40, 95% CI [0.37, 0.43], *p* < .001, Table 2). Additionally, when controlling for baseline emotional and behavioral problems, the association attenuated but remained (β = 0.33, 95% CI [0.30, 0.37], *p* < .001, Table 2). All measured subdomains of executive functioning (inhibition, shifting, emotional control, working memory, and planning/organization) were separately associated with ASD traits in all unadjusted and adjusted models (Table 2).

**Table 2:** The association between executive functioning and ASD traits (n = 3938)

	Mother-reported ASD traits								
	Model 1			Model 2			Model 3		
	β	95% CI	*p*	β	95% CI	*p*	β	95% CI	*p*
Executive functioning total	0.45	0.43–0.48	< .001	0.40	0.37–0.43	< .001	0.33	0.30–0.37	< .001
Inhibition	0.38	0.35–0.41	< .001	0.31	0.28–0.35	< .001	0.22	0.19–0.26	< .001
Shifting	0.33	0.30–0.36	< .001	0.29	0.26–0.32	< .001	0.22	0.19–0.25	< .001
Emotional control	0.30	0.26–0.33	< .001	0.27	0.23–0.30	< .001	0.17	0.14–0.20	< .001
Working memory	0.41	0.38–0.44	< .001	0.34	0.31–0.38	< .001	0.27	0.23–0.30	< .001
Planning/organizing	0.36	0.33–0.39	< .001	0.29	0.26–0.33	< .001	0.21	0.18–0.24	< .001

Parameter estimates are standardized betas with 95% confidence intervals and significance values. Model 1 is unadjusted

Model 2 is adjusted for covariates: gender, gestational age, ethnicity, age at ASD traits questionnaire, maternal education, and maternal psychopathology. Model 3 is adjusted for the covariates in model 2 and baseline emotional and behavioral problems (parent-rated CBCL total problems at age 3)

These findings are generally consistent with the association between executive functioning and ASD diagnosis. More executive functioning problems at age 4 were associated with an almost threefold increase in the odds of having an ASD diagnosis (OR_adjusted_ = 2.92, 95% CI [2.19, 3.89], *p* < .001, Table 3). When controlling for baseline emotional and behavioral problems, the association remained (OR = 2.71, 95% CI [1.91, 3.79], *p* < .001, Table 3). Moreover, impaired inhibition, shifting, emotional control, and working memory were associated with a higher chance of an ASD diagnosis (Table 3). However, after controlling for baseline emotional and behavioral problems, planning was no longer associated with the likelihood of an ASD diagnosis (Table 3).

**Table 3:** The association between executive functioning and ASD diagnoses (n = 3796; diagnoses n = 56)

	ASD diagnoses								
	Model 1			Model 2			Model 3		
	OR	95% CI	*p*	OR	95% CI	*p*	OR	95% CI	*p*
Executive functioning total	3.22	2.49–4.18	< .001	2.90	2.18–3.86	< .001	2.69	1.92–3.77	< .001
Inhibition	5.50	3.58–8.48	< .001	4.25	2.68–6.74	< .001	3.35	1.98–5.67	< .001
Shifting	10.29	6.10–17.37	< .001	7.87	4.56–13.56	< .001	6.39	3.57–11.45	< .001
Emotional control	6.95	4.07–11.86	< .001	5.54	3.15–9.74	< .001	4.13	2.17–7.85	< .001
Working memory	4.72	3.11–7.17	< .001	3.74	2.38–5.90	< .001	2.86	1.72–4.76	< .001
Planning/organizing	4.39	2.39–8.04	< .001	3.04	1.59–5.82	.001	1.81	0.88–3.72	.107

Parameter estimates are odds ratios with 95% confidence intervals and significance values. Model 1 is unadjusted

Model 2 is adjusted for covariates: gender, gestational age, ethnicity, maternal education, and maternal psychopathology. Model 3 is adjusted for the covariates in model 2 and baseline emotional and behavioral problems (parent-rated CBCL total problems at age 3)

### Executive functioning and ADHD traits

More problems in executive functioning at age 4 were associated with more ADHD traits at a later age (β_adjusted_ = 0.38, 95% CI [0.34, 0.41, *p* < .001, Table 4). When controlling for baseline emotional and behavioral problems, the association remained (β = 0.32, 95% CI [0.28, 0.35], *p* < .001, Table 4). Impairment in each subdomain of executive functioning was associated with more ADHD traits, except for emotional control and shifting. Moreover, shifting had a negative association with executive functioning, indicating that more difficulties in this domain were associated with fewer ADHD traits (β_adjusted_ = − 0.11, 95% CI [− 0.15, 0.07], *p* < .001, Table 4).

**Table 4:** The association between executive functioning and ADHD traits (n = 2749)

	Teacher-reported ADHD traits								
	Model 1			Model 2			Model 3		
	β	95% CI	*p*	β	95% CI	*p*	β	95% CI	*p*
Executive functioning total	0.18	0.14–0.22	< .001	0.12	0.08–0.16	< .001	0.12	0.07–0.16	< .001
Inhibition	0.25	0.21–0.29	< .001	0.20	0.16–0.24	< .001	0.21	0.16–0.25	< .001
Shifting	− 0.03	− 0.07–0.01	.108	− 0.07	− 0.11 to − 0.03	< .001	− 0.11	− 0.15 to − 0.07	< .001
Emotional control	0.04	0.003− 0.08	.037	0.02	− 0.02–0.06	.272	-0.01	− 0.05–0.03	.657
Working memory	0.21	0.17− 0.25	< .001	0.15	0.11–0.19	< .001	0.15	0.11–0.19	< .001
Planning/organizing	0.17	0.13− 0.20	< .001	0.11	0.07–0.15	< .001	0.09	0.05–0.14	< .001

Parameter estimates are standardized betas with 95% confidence intervals and significance values. Model 1 is unadjusted

Model 2 is adjusted for covariates: gender, gestational age, ethnicity, age at teacher-reported ADHD traits questionnaire, maternal education, and maternal psychopathology. Model 3 is adjusted for the covariates in model 2 and baseline emotional and behavioral problems (parent-rated CBCL total problems at age 3)

These results are generally consistent with the analyses with ADHD diagnosis as outcome. More executive functioning difficulties at age 4 were associated with a nearly threefold increase in the odds of having ADHD at a later age (OR_adjusted_ = 2.83, 95% CI [2.37, 3.38], *p* < .001, Table 5). When controlling for baseline emotional and behavioral problems, the association remained (OR = 2.32, 95% CI [1.89, 2.85], *p* < .001, Table 5). Additionally, all subdomains of executive functioning were associated with a higher chance of an ADHD diagnosis at a later age, except shifting. Shifting was no longer significant when adjusting for covariates and emotional and behavioral problems (Table 5).

**Table 5:** The association between executive functioning and ADHD diagnoses (n = 4000; diagnoses n = 194)

	ADHD diagnoses								
	Model 1			Model 2			Model 3		
	OR	95% CI	*p*	OR	95% CI	*p*	OR	95% CI	*p*
Executive functioning total	3.18	2.70–3.74	< .001	2.83	2.37–3.38	< .001	2.32	1.89–2.85	< .001
Inhibition	7.33	5.59–9.61	< .001	6.15	4.61–8.20	< .001	4.59	3.34–6.32	< .001
Shifting	3.10	2.29–4.20	< .001	2.30	1.67–3.17	< .001	1.37	0.97–1.95	.077
Emotional control	5.57	4.10–7.55	< .001	4.24	3.08–5.84	< .001	2.59	1.81–3.71	< .001
Working memory	4.73	3.68–6.08	< .001	3.82	2.92–4.99	< .001	2.64	1.96–3.56	< .001
Planning/organizing	7.73	5.46–10.93	< .001	5.74	3.97–8.30	< .001	3.56	2.38–5.33	< .001

Parameter estimates are odds ratios with 95% confidence intervals and significance values. Model 1 is unadjusted

Model 2 is adjusted for covariates: gender, gestational age, ethnicity, age at mother-reported ADHD symptoms interview, maternal education, and maternal psychopathology. Model 3 is adjusted for the covariates in model 2 and baseline emotional and behavioral problems (parent-rated CBCL total problems at age 3)

**Fig. 2:**
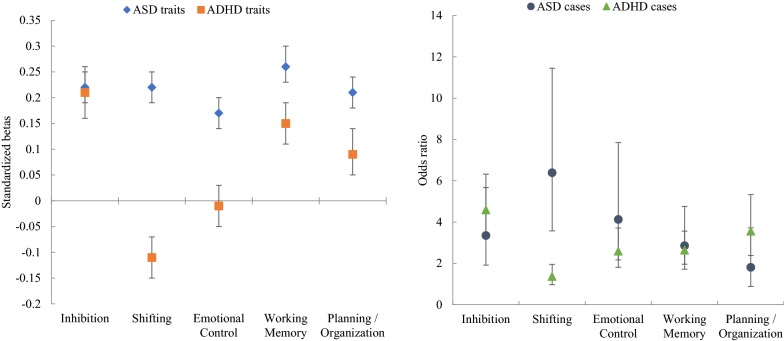
Standardized betas and odds ratios for the relation of executive functioning subscales with ASD and ADHD traits, adjusted for covariates and baseline emotional and behavioral problems (parent-rated CBCL total problems at age 3)

To easily compare the results on ASD and ADHD, Fig. 2 shows the standardized betas for ASD and ADHD traits and odds ratios for ASD and ADHD diagnosis. Sensitivity analysis excluding children with an ASD or ADHD diagnosis indicated similar results, although slightly attenuated (see Additional file 1: Tables S2, S3). When controlling only for baseline ASD traits or ADHD traits in the respective analyses rather than all emotional and behavioral problems, results remained similar, except for planning and ASD diagnosis (OR = 2.01, 95% CI [1.02, 3.98], *p* = .045) and for shifting and ADHD diagnosis (OR = 1.82, 95% CI [1.31, 2.53], *p* < .001).

## Discussion

This study found that impaired executive functioning at the age of 4 years was prospectively associated with ASD and ADHD traits 2–3 years later, independent of multiple confounders and pre-existing psychopathology. Difficulties across executive functioning domains were associated with higher levels of ASD traits, whereas only impaired inhibition, working memory, and planning/organization were associated with more traits of ADHD. Importantly, our findings were consistent across informants: mother-reported ASD traits and clinical ASD diagnoses yielded similar results, as did teacher-reported ADHD traits and ADHD diagnoses based on mother reports. When excluding children with an ASD or ADHD diagnosis from the analysis, we were able to confirm that this association is not fully driven by a subgroup with clinically relevant levels of ASD and ADHD traits, but that, importantly, the associations were also observed in children with sub-clinical levels of these traits. Therefore, our findings provide evidence for a graded association of executive function impairments along the continuum of ASD and ADHD. Due to the nature of our data, we cannot draw any causal conclusions. However, our results implicate future studies to add to our findings, examining the causality of this relationship more in depth.

In line with several previous studies [5, 19, 20, 25], we found that difficulties in all subdomains of executive functioning were associated with higher levels of ASD traits as well as a greater risk of having an ASD diagnosis. Some studies suggest that deficits primarily in shifting and planning characterize ASD [5, 6], and that these domains distinguish children with ASD from children with other developmental disorders. Our findings do suggest that shifting may be more predictive for clinical ASD than other executive functioning domains, which might be explained by the high resemblance to the rigid and inflexible behavioral patterns characterizing ASD [7].

Our study also showed that deficits in overall executive functioning were associated with higher levels of ADHD traits and with a greater likelihood of being diagnosed with ADHD. In line with most previous research, specific domains of executive functioning, inhibition, working memory, and planning/organization, were related to ADHD traits and likewise to ADHD diagnoses [17, 18]. However, not all studies found planning to be impaired in children with ADHD [5, 65]. This could be due to the different ways of measuring planning (performance task or behavioral rating). Interestingly, we found that better shifting abilities were related to higher levels of ADHD traits. Perhaps teachers mistook the child’s ability to easily switch between situations for inattention. This association was, however, not significant for ADHD cases in this study, and has not been described previously [5, 17]. Further exploration and replication of our finding is needed.

The results of the current study support the notion that executive functioning deficits overlap considerably among neurodevelopmental disorders. A general psychopathology factor has indeed been identified by multiple studies [66, 67], suggesting a substantial phenomenological overlap among (neurodevelopmental) psychopathology. The association of executive functioning with the general psychopathology factor was similar to the relation between executive functioning and separate disorders [68, 69]. This is supported by several previous studies, which have proposed that problems in executive function constitute an important part of the broader phenotypes of ASD and ADHD [23, 70, 71]. Furthermore, polygenic risk studies have shown that clinical and subclinical ASD and ADHD share latent genetic vulnerability [42]. Also, neuroimaging studies observed that frontal areas in the brain are involved in the development of ASD and ADHD symptoms, such as hypoactivation in frontal and parietal regions [52, 72–74], and similar brain areas are implicated in executive functioning [75]. All this possibly indicates that an underlying factor contributes to executive functioning, ASD, and ADHD.

Despite this evidence for an overlap of executive functioning deficits with ASD and ADHD symptoms, unique variance needs to be considered as well. Reviews on the neurobiology of ASD and ADHD show several differences [73, 74], such as deficient connectivity between networks in the brain, which shows stronger association with ASD, and deficits in the attentional network, which has stronger associations with ADHD. These specific underlying neural correlates could potentially explain the differing patterns of associations of executive functioning deficits with ASD and ADHD traits that were found in the current and other studies [5, 16, 17], as well as differences in behavioral expression. Additionally, various unique genetic influences for ASD and ADHD have been found in twin and molecular studies [76–78], which might also explain differences in behavior between these disorders. Reviewing the evidence for unique and overlapping variance among executive dysfunction, ASD, and ADHD, a combination of specific and shared factors is likely to be most accurate: an underlying construct may explain similarities in the areas of executive functioning deficits, ASD, and ADHD, yet each problem domain results from unique genetic, neurobiological and environmental contributing factors, which, in turn, lead to differential behavioral expressions. More research is needed on the similarities and differences among executive functioning and neurodevelopmental problems, and what role executive functioning plays in their etiologies.

Executive dysfunction could be part of the broader phenotype of neurodevelopmental traits, but our findings also suggest other possibilities. The longitudinal design of this study suggests some developmental difference in the trajectory of symptoms: rather than being parallel to ASD and ADHD traits, executive functioning may precede traits of these neurodevelopmental disorders. The associations remained even after adjusting for baseline behavioral problems. It could potentially be that deficits in executive functioning worsen the expression of children’s ASD or ADHD traits and, reversely, perhaps good executive functioning skills can serve as a buffer, tempering the severity of developmental disorders [79]. However, a more likely explanation is that problems in executive functioning are an expression of the latent genetic vulnerability for ASD and ADHD [42].

### Strengths and limitations

The current study had several strengths. First, we examined the prospective relationship between executive functioning and neurodevelopmental disorders in very young children in a large cohort, enabling us to control for multiple confounding variables, importantly baseline emotional and behavioral problems of the children. Second, we used multiple informants in this study; namely mothers, teachers, and medical records, yielding largely consistent results across these raters. Finally, both clinical diagnoses as well as sub-threshold traits of ASD and ADHD were considered, which addresses the research questions across the neurodevelopmental continuum.

Despite these strengths, multiple limitations need to be mentioned as well. First, the non-response analysis indicated that socially disadvantaged children who are at higher risk of psychiatric problems were more likely to drop out. However, this selective loss to follow-up seems to affect only prevalence estimates, while longitudinal relationships estimated by association analyses remain relatively unchanged [80]. Second, despite our careful approach to identify those likely to have an ASD or ADHD diagnosis, we potentially missed cases. We also lack the data of diagnosis of ASD, as the children were likely diagnosed within the first 2 or 3 years of life. Third, we measured executive functioning with the BRIEF-P, a questionnaire that was completed mostly by mothers. Despite the marginal but considerable correlation between informants, it is recommended to verify whether the results remain with different informants [45]. Last, most of our questionnaires were completed by mothers, inducing considerable shared method variance. Nonetheless, to address this, the TRF to assess ADHD traits was administered to teachers and the ASD diagnoses were verified by medical records.

## Conclusions

Our findings suggest that early executive functioning impairments may be a precursor of neurodevelopmental problems at a later age, for both children with clinical as well as with sub-clinical traits of ASD and ADHD. This supports the idea that children in the sub-clinical range should not be forgotten, but rather should be able to receive help when needed. Moreover, although it is not our aim to propose changes to the diagnostic framework, our results could point towards a possibility of identifying and monitoring children early who are at risk for developing clinical ASD or ADHD or having greater severity of ASD or ADHD. This allows for early intervention, which can potentially help prevent children from having persisting difficulties in executive function, developing more severe neurodevelopmental problems, and having negative outcomes later in life.

## Supplementary information

**Additional file 1: Table S1.** Correlations Between Predictor and Outcome Variables. **Table S2.** The Association Between Executive Functioning and ASD Traits After Removing Clinical Cases (n = 3731). **Table S3.** The Association Between Executive Functioning and ADHD Traits After Removing Clinical Cases (n = 2612).

## Data Availability

The datasets analyzed during the current study are not publicly available due to the terms and conditions participants agree to when they participate in Generation R, but are available from the corresponding author on reasonable request.

## Abbreviations

ADHD: attention-deficit/hyperactivity disorder
ASD: autism spectrum disorder
BRIEF-P: Brief Rating Inventory of Executive Function-Preschool Version
BSI: Brief Symptom Inventory
CBCL: Child Behavior Checklist
SCQ: Social Communication Questionnaire
SES: socio-economic status
SRS: Social Responsiveness Scale
TRF: Teacher Report Form
