# Supplementary material for: Executive functioning and neurodevelopmental disorders in early childhood: a prospective population-based study
Source: Child Adolesc Psychiatry Ment Health. 2019 Oct 22;13:38. doi: 10.1186/s13034-019-0299-7 (PMC6805591; doi:10.1186/s13034-019-0299-7)
Supplement: Supplementary file 1 — Additional file 1: Table S1. Correlations Between Predictor and Outcome Variables. Table S2. The Association Between Executive Functioning and ASD Traits After Removing Clinical Cases (n = 3731). Table S3. The Association Between Executive Functioning and ADHD Traits After Removing Clinical Cases (n = 2612). [file 13034_2019_299_MOESM1_ESM.docx]

Table S1. Correlations Between Predictor and Outcome Variables

|  | 1. | 2. | 3. | 4. | 5. | 6. | 7. | 8. | 9. |
| --- | --- | --- | --- | --- | --- | --- | --- | --- | --- |
| 1. Executive functioning – inhibition | 1 |  |  |  |  |  |  |  |  |
| 2. Executive functioning – shifting | .32** | 1 |  |  |  |  |  |  |  |
| 3. Executive functioning – emotional control | .56** | .52** | 1 |  |  |  |  |  |  |
| 4. Executive functioning – working memory | .71** | .35** | .43** | 1 |  |  |  |  |  |
| 5. Executive functioning – planning / organization | .66** | .32** | .42** | .76** | 1 |  |  |  |  |
| 6. Executive functioning – overall ability | .87** | .61** | .73** | .86** | .81** | 1 |  |  |  |
| 7. ASD traits | .37** | .32** | .29** | .40** | .34** | .45** | 1 |  |  |
| 8. ADHD traits – teacher-reported | .19** | -.06* | .02 | .14** | .11** | .12** | .16** | 1 |  |
| 9. ADHD symptoms – mother-reported | .45** | .08* | .22** | .34** | .31** | .37** | .38** | .40** |  |

*Note.* **p* < .05. ***p* < .01. **Executive functioning was mother-rated and assessed at age 4. ASD traits were reported by mothers at age 6. ADHD traits were teacher-reported at age 7. ADHD symptoms were reported by mothers at age 7.**

Table S2. The Association Between Executive Functioning and ASD Traits After Removing Clinical Cases (n = 3731)

|  | Mother-Reported ASD Traits | | | | | | | | | | | | | | |
| --- | --- | --- | --- | --- | --- | --- | --- | --- | --- | --- | --- | --- | --- | --- | --- |
|  | Model 1 | | |  | | Model 2 | | | |  | | Model 3 | | | |
|  | β | 95*%* CI | *p* |  | β | | 95*%* CI | *p* |  | | β | | 95*%* CI | *p* |  |
| Executive functioning total | 0.40 | 0.37 – 0.43 | <.001 |  | 0.35 | | 0.32 – 0.39 | <.001 |  | | 0.29 | | 0.25 – 0.32 | <.001 |  |
| Inhibition | 0.32 | 0.29 – 0.35 | <.001 |  | 0.26 | | 0.23 – 0.29 | <.001 |  | | 0.18 | | 0.14 – 0.21 | <.001 |  |
| Shifting | 0.28 | 0.25 – 0.31 | <.001 |  | 0.26 | | 0.23 – 0.29 | <.001 |  | | 0.19 | | 0.16 – 0.22 | <.001 |  |
| Emotional control | 0.25 | 0.21 – 0.28 | <.001 |  | 0.23 | | 0.20 – 0.26 | <.001 |  | | 0.14 | | 0.11 – 0.18 | <.001 |  |
| Working memory | 0.35 | 0.32 – 0.39 | <.001 |  | 0.30 | | 0.27 – 0.33 | <.001 |  | | 0.23 | | 0.19 – 0.26 | <.001 |  |
| Planning/organizing | 0.31 | 0.28 – 0.34 | <.001 |  | 0.26 | | 0.23 – 0.29 | <.001 |  | | 0.19 | | 0.15 – 0.22 | <.001 |  |

*Note.* Parameter estimates are standardized betas with 95*%* confidence intervals and significance values. Model 1 is unadjusted.
Model 2 is adjusted for covariates: gender, gestational age, ethnicity, age at ASD traits questionnaire, maternal education, and maternal psychopathology. Model 3 is adjusted for the covariates in model 2 and baseline behavioral problems **(parent-rated CBCL total problems at age 3)**.

Table S3. The Association Between Executive Functioning and ADHD Traits After Removing Clinical Cases (n = 2612)

|  | Teacher-Reported ADHD Traits | | | | | | | | | | | | | | |
| --- | --- | --- | --- | --- | --- | --- | --- | --- | --- | --- | --- | --- | --- | --- | --- |
|  | Model 1 | | |  | | Model 2 | | | |  | | Model 3 | | | |
|  | β | 95*%* CI | *p* |  | β | | 95*%* CI | *p* |  | | β | | 95*%* CI | *p* |  |
| Executive functioning total | 0.15 | 0.11 – 0.19 | <.001 |  | 0.09 | | 0.05 – 0.14 | <.001 |  | | 0.09 | | 0.04 – 0.14 | <.001 |  |
| Inhibition | 0.22 | 0.18 – 0.26 | <.001 |  | 0.17 | | 0.13 – 0.21 | <.001 |  | | 0.18 | | 0.13 – 0.22 | <.001 |  |
| Shifting | -0.06 | -0.10 - -0.02 | .005 |  | -0.09 | | -0.13 - -0.05 | <.001 |  | | -0.12 | | -0.16 - -0.08 | <.001 |  |
| Emotional control | 0.01 | -0.03 – 0.05 | .557 |  | 0.002 | | -0.04 – 0.04 | .923 |  | | -0.03 | | -0.07 – 0.02 | .259 |  |
| Working memory | 0.19 | 0.15 – 0.24 | <.001 |  | 0.14 | | 0.10 – 0.18 | <.001 |  | | 0.14 | | 0.09 – 0.18 | <.001 |  |
| Planning/organizing | 0.14 | 0.10 – 0.18 | <.001 |  | 0.09 | | 0.05 – 0.13 | <.001 |  | | 0.08 | | 0.04 – 0.12 | <.001 |  |

*Note.* Parameter estimates are standardized betas with 95*%* confidence intervals and significance values. Model 1 is unadjusted.
Model 2 is adjusted for covariates: gender, gestational age, ethnicity, age at ADHD traits questionnaire, maternal education, and maternal psychopathology. Model 3 is adjusted for the covariates in model 2 and baseline behavioral problems **(parent-rated CBCL total problems at age 3)**.
